# Pediatric injury due to wheeled recreational devices: a single-institution retrospective study

**DOI:** 10.1186/s40621-022-00395-5

**Published:** 2022-12-21

**Authors:** John Charles Nichols, Annalise Sorrentino, Margaret Hayslip, William King, Angela Jones, Kathy Monroe

**Affiliations:** 1 University of Alabama School of Medicine, 1670 University Blvd, Birmingham, AL 35233 USA; 2 Children’s of Alabama University of Alabama Birmingham, 1600 7th Ave So, Suite 110 CPP, Children’s of Alabama, Birmingham, AL 35233 USA; 3 Medical Informatics, Children’s of Alabama University of Alabama Birmingham, 1600 7th Ave So, Suite 110 CPP, Children’s of Alabama, Birmingham, AL 35233 USA; 4 Division of Pediatric Emergency Medicine University of Alabama Birmingham, Department of Pediatrics, University of Alabama School of Medicine, 1600 7th Ave So, Suite 110 CPP, Children’s of Alabama, Birmingham, AL 35233 USA

**Keywords:** Wheeled recreational device (WRD), Injury prevention, Pediatric, Helmet use, Bicycle, ATV

## Abstract

**Background:**

Injuries are the number one cause of death in children and cause significant morbidity. Common scenarios for injury include wheeled recreational devices (WRDs) that allow children to be mobile and independent (example ATV-all terrain vehicles, dirt bikes, bicycles, skateboards, and scooters). We present a case series review of these external causes of injury. This study aims to evaluate epidemiologic trends in WRD injuries and patterns in usage of protective gear.

**Results:**

A total of 263 patients were identified as meeting criteria for inclusion with the following causes of injuries-103 bicycle, 73 ATV, 27 dirt bike, 14 skateboard, 13 motorcycle, 7 go carts, 3 hover board, 3 roller skates, 1 dune buggy, 1 motor scooter, 1 rip stick, and 1 tractor toy. Ages of patients ranged from 2 to 18 years of age with the greatest range being noted for bicycles (2–17 years) and motorcycles (3–18 years). The mean age was higher for motorcycle and skateboard (12.9 and 11.6, respectively) and lowest for scooter and bicycle (8.3 and 9.2, respectively). The majority of [overall study (97%) and for each mode of transportation] patients were Caucasian, which is in contrast to our overall ED population, which is only 42% Caucasian. The majority of patients were male [190 (72%)]. Over half of the overall patients, 159 of the 263 (60%), were not wearing a helmet (with only 10 charts having no documentation of helmet use). In regard to ATV riders specifically, 58% were not wearing helmets at the time of injury, with an additional 5 patients who reported their helmet came off. The lowest percentage of riders reporting appropriate helmet use was skateboarders with only 21% wearing helmets, and the highest percentage was dirt bike riders with 74% reporting helmet use.

**Conclusion:**

Common scenarios for injury include WRDs that allow children to be mobile and independent. Most of these injuries were found in Caucasian males between the ages of 9–12 with low rates of helmet use. This study adds to the literature with a description of the breadth of products children use and are injured while using.

**Supplementary Information:**

The online version contains supplementary material available at 10.1186/s40621-022-00395-5.

## Background

Unintentional injury is the leading cause of death among the pediatric population greater than 1 year of age (MedlinePlus, U.S. National Library of Medicine 2021). While motor vehicle collisions make up the majority of these accidents, unintentional recreational and sports injuries contribute considerably. Additionally, unintentional fall is the first leading cause of non-fatal injury in the pediatric population, with wheeled recreational devices making up a substantial portion of injury (Kaddis et al. 2016; Centers for Disease Control and Prevention 2019).

Injuries from recreational devices are a common reason for presentation to the pediatric emergency department. Protective equipment has been proven to decrease the severity of injuries, yet there are still many patients who do not use any. While there have been studies on certain subsects of wheeled recreational devices (WRDs), no studies to date have encompassed all wheeled recreational devices leading to injuries presenting in an urban pediatric emergency department.

A recent study also found that injuries involving non-motorized wheeled recreational devices and bicycles were a common reason for presentation to the emergency department. Helmet use has been shown to reduce mortality and morbidity caused by head injury, but there is still a significant portion of users who do not wear helmets or other protective gear (Ong et al. 2018). Reinforcing safety measures on wheeled recreational devices can help decrease injury severity and presentation to the emergency department.

The objective of this chart review is to evaluate traumas caused by WRDs and observe how protective gear and environmental factors may affect injury. WRDs involve any device containing wheels that is used for leisure and independent mobility. WRDs included in our review include bicycles, ATVs, dirt bikes, scooters, skateboards, motorcycles, go carts, hoverboards, roller skates, rip sticks, and tractor toys.

## Results

During the study period (1/1/19–12/31/19), 2,062 injuries due to external causes presented to Children’s of Alabama ED, with 263 meeting study criteria. Therefore, 12.8% of injuries with external cause seen during 2019 were due to WRD use. Patient ages in this study ranged from 2 to 18 years, and the overall median age was 11 years.

### Overall study demographic data compared to all injuries

The demographic data of pediatric injuries due to WRD differed considerably from the ED population, with WRD injuries occurring most frequently in older white males. As seen in Table 1, the vast majority of the patients in this study were white (79.1%, *n* = 208), with only 18.25% (*n* = 48) black and 2.65% (*n* = 7) other or undetermined race. There is a statistically significant higher proportion (32.3%) of white patients injured due to WRDs (95% CI 26.9%, 36.9%) when compared to the proportion of white patients for all-cause injuries (46.8%).

**Table 1:** Body parts injured

Most common body parts injured (*N* = 1333)		
Body part	Frequency (*n*)	% Total
Head or face	329	24.7
Wrist or hand	254	19.1
Ankle or foot	208	15.6
Lower limb	188	14.1
Upper limb	149	11.2
Shoulder	81	6.1
Trunk	60	4.5
Other	44	3.3
Neck	19	1.4
Genital	1	0.1

Similarly, Table 1 also demonstrates that the proportion of WRD injuries occurring in males (72.2%) was significantly greater than the proportion of all-cause injuries occurring in males (56.9%), with a difference in proportions of 22.2% (95% CI 16.8%, 26.7%). A statistically significant difference in mean ages between WRD injuries (10.5 years) and all-cause injuries (7.1 years) was also observed, with a 3.4 years higher mean age for WRD injuries (95% CI 3.0, 3.8).

### WRD frequency of injury

**Fig. 1:**
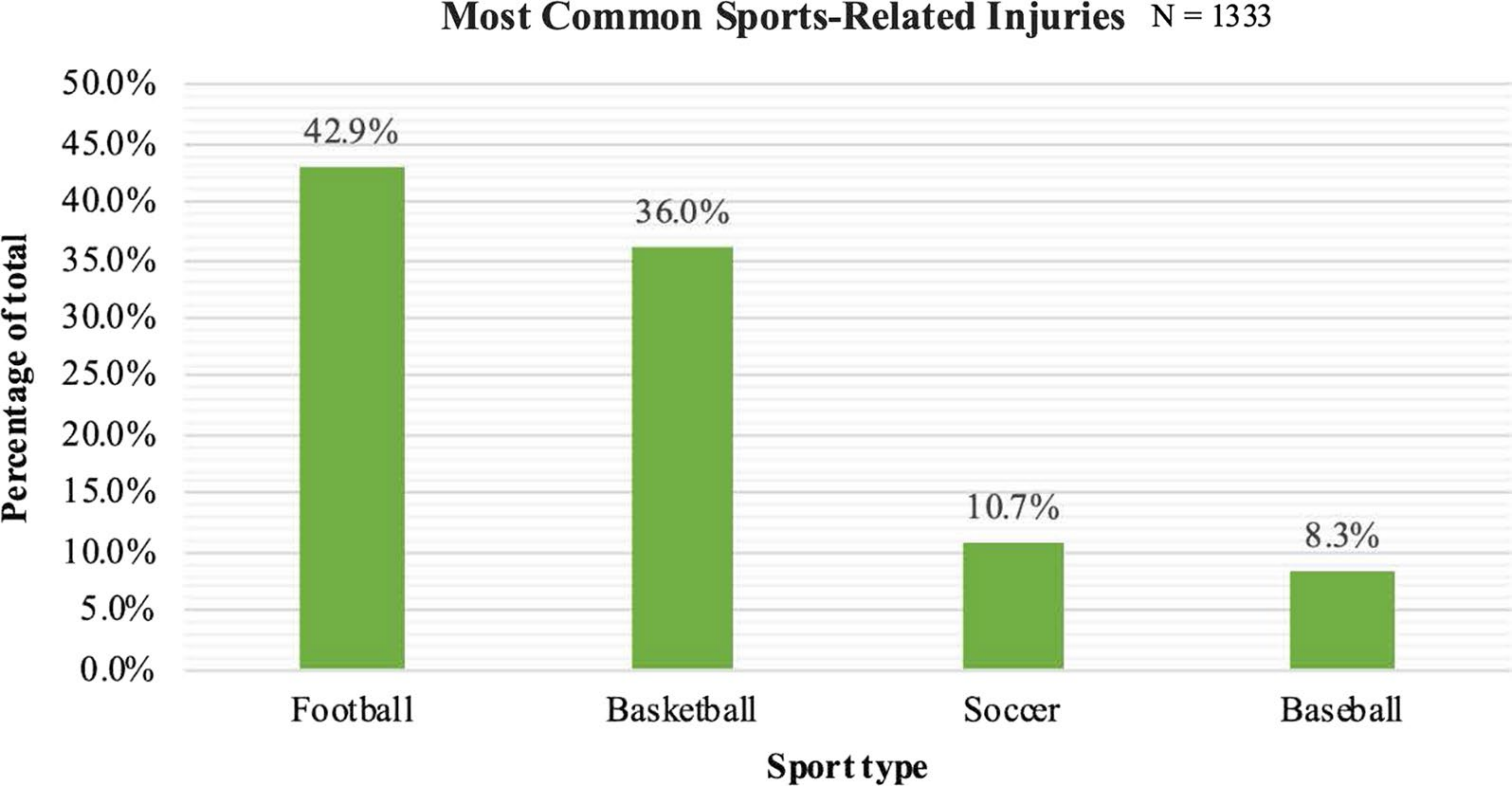
Most common sports causing pediatric SRIs

Bicycles and all-terrain vehicles (ATVs) comprised two-thirds of the total WRD injuries when considered together (39.7%, *n* = 103 and 27.8%, *n* = 73, respectively). Injury frequencies for all other vehicle types were much lower, with 9.9% of injuries accounted by dirt bikes (*n* = 26), 6.1% resulting from scooters (*n* = 16), 5.3% resulting from skateboards (*n* = 14), and 4.9% resulting from motorcycles (*n* = 13). The relative frequencies of each of the six major vehicle types leading to injury are displayed in Fig. 1. The remaining 7.0% of injuries (*n* = 17) resulted from use of go carts (*n* = 7), hoverboards (*n* = 3), roller skates (*n* = 3), dune buggy (*n* = 1), motor scooter (*n* = 1), rip stick (*n* = 1), and tractor toy (*n* = 1).

### Injury-specific data by vehicle type

General data for all WRD injuries by vehicle type are listed in Table 2. The largest age ranges were noted for bicycle and motorcycle injuries (15 years each), with the highest mean ages noted in roller skating (14) and motorcycle (12.9). The majority of injuries for all vehicle types except rip stick and roller skating (which both had small sample sizes) occurred in males, with the highest proportions of males injured observed in dirt bike (96.3%) and skateboard (92.6%), excluding vehicle types with *n* < 10.

**Table 2:** Modes of injury and organization of play

Frequency of injury mode and organization (*n* = 1333)		
Mode of injury	Frequency	% of Total
Direct/contact injury	723	54.3
Indirect/non-contact injury	583	43.7
Not documented	27	2.0
Organization of play		
Organized	804	60.3
Unorganized	432	32.4
Not documented	97	7.3

As listed in Table 2, admission was significantly more common in ATV injuries (41.1%, *n* = 30) compared with bicycle injury (16.5%, *n* = 17), with a 24.6% difference in admission rates (95% CI 10.3%, 38.3%). Although the admission rate for ATV injuries was also higher than that of dirt bike injuries (25.9%), this difference is not statistically significant. Admission rates for other vehicles were not compared because of the small sample sizes. However, a statistically significant difference in mean age of 1.3 years (95% CI 0.3, 2.4) was observed between patients who were admitted (11.5) and discharged (10.2), with admissions occurring more commonly in slightly older patients. The percentage of patients who were noted not to be wearing protective head gear is also displayed in Table 2.

### Helmet usage

**Fig. 2:**
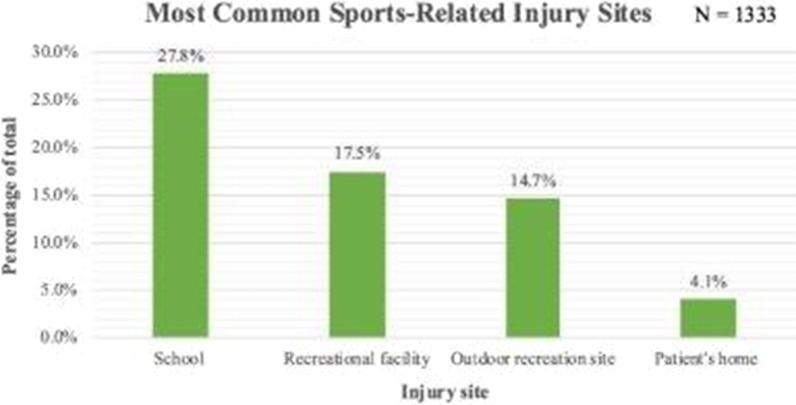
Most common sports-related injury sites

When considering all WRD-related injuries, 39.5% (*n* = 104) of patients were wearing helmets at the time of injury, with 18 cases not documented. As displayed in Fig. 2, only two of the six vehicle types with the highest proportions of injuries were found to have a helmet usage rate of greater than 50.0%, with 74.1% of dirt bike users and 69.2% of motorcycle users wearing helmets. Less than half of the patients using the other four major vehicle types were wearing helmets, with helmet use reported in 42.5% of ATV injuries, 37.9% of bicycle injuries, 31.3% of scooter injuries, and only 21.4% of skateboard injuries. The remaining 7.0% of WRD injuries were excluded from the chart, due to small sample size, but helmet data regarding these vehicle types can be seen in Table 2.

When comparing helmet usage across race categories, a significantly higher proportion of white patients used helmets (47.4%) compared to nonwhite patients (18.9%), with a difference in helmet rates of 28.5% (95% CI 14.3%, 39.4%). However, there were no significant differences in frequency of helmet use by age or gender observed. Additionally, the difference in admission rates for helmeted patients was not statistically significant compared to non-helmeted patients.

## Discussion

This study supports previous findings that children are commonly injured on wheeled recreational devices (Kaddis et al. 2016; Foujuoh et al. 2002; Lindsay and Brussoni 2014). Most studies include skateboard, scooter, inline skating, rollerblades and some include bicycles. We included all these with the addition of ATVs. While ATVs were not originally designed for recreation, they have become popular as recreational vehicles. Many families ride ATVs together and many children ride adult-sized ATVs alone or with other children, and children are frequently injured while riding on these vehicles (Denning et al. 2013; Garay et al. 2017). In fact, children who ride ATVs are at a twelve times greater risk of injury compared to adults (US Consumer Product Safety Commission 2021). Since these are now commonly used as recreational devices, we have included them in this study.

Our study found median age for children injured on WRD to be 11 years, which is similar to a prior study that did not include bicycles nor ATVs in which the median age was 11 years (Kaddis et al. 2016). One would think that the inclusion of ATVs might actually make our study median age higher as the younger children should not be riding ATVs, but that was not found to be the case. Age ranges were noted to be quite wide for all vehicle types. As can be seen in Table 2, children as young as one, two or three years of age were injured on bicycles, ATVs, hoverboards, and motorcycles. None of these WRDs are meant for children of that age. These injuries were incurred as passengers, and this highlights a need for education about safety regarding these devices. The vast majority of patients in this study were white (79%) despite the racial mix of our overall ED to be 47% white. This may indicate differences in access to these type vehicles. Males accounted for more injuries due to WRDs (72%), which is similar to prior studies (Foujuoh et al. 2002; Lindsay and Brussoni 2014).

Bicycles and all-terrain vehicles (ATVs) comprised two-thirds of the total WRD injuries when considered together (39.16%, *n* = 103 and 27.76%, *n* = 73, respectively). While the recommendations for riding an ATV include age > 16 years, we found many children far younger were injured on these vehicles. The Consumer Product Safety Commission states that youth < 16 years of age have represented approximately one-quarter of all US ATV related injuries, more than any other age-groups (US Consumer Product Safety Commission 2021). A variety of other vehicles accounted for the remainder of the WRDs.

Bicycle and ATV crashes can result in severe traumatic brain injury (Denning et al. 2013; Dellinger and Kresnow 2010). Helmets have been shown to prevent head trauma in both activities, and both ATV and bicycles have helmet recommendations. We found only 39.5% of the overall study population were wearing helmets at the time of the crash, with only 38% of bicycle and 43% of ATV riders reporting wearing a helmet at the time of injury. Previous studies have shown that while head injuries are among the most common injuries sustained in ATV and bike crashes, compliance with helmet use is low (16% in Adil study; 13.5% in Forjuoh Wymore) (Foujuoh et al. 2002; Adil et al. 2017; Wymore et al. 2020). Likewise, helmet use for inline skating, skateboarding, and scooter riding has also been shown to be low (Adil et al. 2017) as we found in our study. In this study, we only addressed whether a helmet was worn or not. We did not assess correct wearing of helmet. Prior studies have shown that prevalence of correct helmet use varies significantly by activity (Foujuoh et al. 2002).

## Limitations

This is a retrospective review of injuries, which is limited by the available data. The rate of documentation for helmet use was extremely high. This may be due to a prior intervention at our institution in which we added helmet use as a hard stop documentation component to the electronic medical record. These findings represent a single southern institution, but do seem similar to prior studies conducted in other areas of the country.

## Conclusions

Consumer Product Safety Commission reported riding toys were associated with 69,400 of the estimated injuries, and 70 percent of which were related to non-motorized scooters in 2019. Our study contributes to the literature by describing one southern institution’s rates of wheeled recreational device-related injury patterns. The age ranges of children injured while using these vehicles are wide with extremely young children, as young as one year of age, being injured. Education to parents must start early before the children are exposed to risks associated with these vehicles. Helmet use remains low for all WRD types. Education and awareness of the potential hazards of these common devices are indicated.

## Methods

### Study design

A retrospective review of WRD injuries presenting to the Children’s of Alabama (COA) Emergency Department between January 1, 2019, and December 31, 2019, was conducted. The electronic medical record was queried using ICD-10 codes to identify patient cases corresponding to injuries due to external causes. The dataset was de-identified and exported to Microsoft Excel. A detailed chart review of this file was conducted by a single team member in order to identify cases meeting inclusion criteria per the history of present illness and other information in the chart. Cases with a potential discrepancy were discussed as a team prior to inclusion or exclusion. This project was deemed exempt by the institutional review board.

### Setting

The COA Emergency Department is the only free-standing level-1 trauma center for children in the state. The annual volume for the ED is greater than 70,000. COA receives children from throughout the State of Alabama.

### Definitions and study criteria

Injuries due to active use of a WRD were included. Wheeled recreational devices (WRDs) are defined in Kaddis et al. to include skateboards, scooters, motorized foot scooters, and rollerblades; however, bicycles and motorized devices were not considered (Kaddis et al. 2016). An alternate study (Collins, et al.) considered exclusively non-automobile motorized vehicle-related injuries (Collins et al. 2007). For the purposes of this study, WRDs were considered as any device designed for operation by individuals, including pediatric patients, that allows for mobility and independence, and for which recreation and transportation are both primary marketed uses. This group includes motorized and non-motorized vehicles, such as bicycles, scooters, skateboards, ATVs, dirt bikes, motorcycles, and other similar devices (Additional file 1: Table S1).

Specific inclusion criteria were as follows: chief complaint was injury due to active use of WRD, presentation to COA Emergency Department (ED) in 2019, and less than 19 years of age. Both passengers and drivers of WRDs were included. For cases meeting study criteria, demographic data (age, gender, race) were recorded, along with injury-specific data (vehicle type, helmet use, site of injury) and data related to the hospital course (disposition, length of stay). Data regarding vehicle type and helmet usage were identified and recorded based on chart review, while all other information was auto-populated from the electronic medical record (EMR).

### Statistical analysis

Data organization and descriptive statistical analyses were conducted using Microsoft Excel and Epi Info 7 (CDC). Statistical comparisons and analyses included: descriptive tabulations and crosstabulations, differences in independent proportions with 95% confidence interval estimations and unpaired t test of means (Welch’s adjustment when appropriate) with 95% confidence interval estimation of the mean differences.

## Supplementary Information

**Additional file 1: Table S1.** Organization of Sport Classification System.

## Data Availability

We are happy to make our data available upon request.

## Abbreviations

ATV: All-terrain vehicle
COA: Children’s of Alabama
ED: Emergency Department
EMR: Electronic medical record
WRD: Wheeled recreational device
