# Supplementary material for: Pediatric injury due to wheeled recreational devices: a single-institution retrospective study
Source: Inj Epidemiol. 2022 Dec 21;9(Suppl 1):44. doi: 10.1186/s40621-022-00395-5 (PMC9768874; doi:10.1186/s40621-022-00395-5)
Supplement: Supplementary file 1 — Additional file 1: Table S1. Organization of Sport Classification System. [file 40621_2022_395_MOESM1_ESM.docx]

| **Organization Classification Criteria** | |
| --- | --- |
| **Characteristic** | **Classification** |
| Game, practice mentioned | Organized |
| Coach, trainer referenced |  |
| Teammate, drills mentioned |  |
| Use of helmet/pads |  |
| Helmet-to-Helmet Contact |  |
| Injured at school outside school hours |  |
| Injury occurred "at/in/during" sport |  |
| School/PE |  |
| "Other player" referenced |  |
| Injured by tackle at rec. facility |  |
| Injury occurred at home of pt or friend | Unorganized |
| Injury occurred in neighborhood |  |
| Basketball injury occurred outdoors |  |
| Injury occurred at detention center |  |
| Inappropriate footwear for sport |  |
| Playing with "friends" |  |

Supplemental Table 1 – Organization of Sport Classification System
